# Neutrophil-to-lymphocyte ratio, calprotectin and YKL-40 in patients with chronic obstructive pulmonary disease: correlations and 5-year mortality – a cohort study

**DOI:** 10.1186/s12950-015-0064-5

**Published:** 2015-03-18

**Authors:** Allan Klitgaard Sørensen, Dennis Back Holmgaard, Lone Hagens Mygind, Julia Johansen, Court Pedersen

**Affiliations:** Department of Infectious Diseases Q, Odense University Hospital, Odense, Denmark; Department of Clinical Microbiology, Hvidovre Hospital, Hvidovre, Denmark; Department of Infectious Diseases, Aalborg University Hospital, Aalborg, Denmark; Departments of Medicine and Oncology, Herlev Hospital, University of Copenhagen, Copenhagen, Denmark

**Keywords:** COPD, Neutrophil-to-lymphocyte ratio, Calprotectin, YKL-40, Lymphopenia, Mortality, Glucocorticoids, Prognosis

## Abstract

**Background:**

Chronic obstructive pulmonary disease (COPD) is characterized by chronic inflammation and progressive decline in pulmonary function. Neutrophil-to-lymphocyte ratio (NLR), YKL-40 and calprotectin are biomarkers of inflammation and predict mortality in patients with different inflammatory diseases. We aimed to investigate the correlation between levels of these three biomarkers and neutrophil granulocyte and lymphocyte count in patients with moderate to very severe COPD stratified by use of systemic glucocorticoids. Furthermore, we studied the ability of these biomarkers to predict all-cause mortality.

**Methods:**

386 patients with moderate to very severe COPD were followed prospectively for 10 years. Patients were divided into two groups according to systemic glucocorticoid use at baseline. Correlations between biomarkers were assessed by Spearman’s Rho, and mortality was evaluated in uni- and multivariate Cox regression analyses with hazard ratios (HR) and 95% confidence intervals (CI).

**Results:**

Plasma calprotectin was positively correlated with neutrophil granulocyte count and NLR. No significant association was found between plasma YKL-40 and the cellular biomarkers, irrespective of glucocorticoid treatment. In the group not treated with systemic glucocorticoids, plasma calprotectin [HR 1.002 (95% CI 1.000 – 1.004)], NLR [HR 1.090 (1.036 – 1.148)] and lymphocyte count [HR 0.667 (0.522 – 0.851)] were significantly associated with higher mortality. In the group treated with systemic glucocorticoids, higher plasma YKL-40 was significantly associated with mortality in univariate Cox regression analysis [HR 1.006 (1.003 – 1.008)].

**Conclusions:**

Calprotectin was related to neutrophil granulocyte count and NLR in patients with moderate to very severe COPD in stable phase and not in treatment with systemic glucocorticoids. Lymphopenia, higher plasma calprotectin and higher NLR were independent predictors of increased all-cause mortality in this group. Our data also suggests that treatment with systemic glucocorticoids has a significant impact on the ability of inflammatory biomarkers to predict all-cause mortality.

**Trial registration:**

ClinicalTrials.gov NCT00132860.

## Introduction

Chronic obstructive pulmonary disease (COPD) is a disease characterized by progressive destruction of lung tissue resulting in a decline in pulmonary function [1]. The pathogenesis of COPD is complex. It is generally accepted that the inflammatory response associated with prolonged exposure to noxious gases like tobacco smoke plays an important role. The pulmonary inflammatory response is accompanied by a chronic low grade systemic inflammatory response [2]. The course of disease progression in patients with COPD is very heterogeneous [3], and this could be due to differences in the level of inflammation. One widely used method of prognosis assessment is the BODE index, which compiles a panel of known predictors of mortality among COPD patients, i.e. low body mass index (BMI), low forced expiratory volume in 1 second (FEV_1_), level of dyspnea and exercise capacity, into an index which is associated with increased risk of mortality [4]. None of these variables directly reflect the degree of inflammation. Inflammatory biomarkers may help to identify a subgroup of COPD patients with a higher level of basal inflammation and with a more rapid disease progression [5]. In a study by Celli et al., it was found that the ability of clinical variables – BODE, age and hospitalization history – to predict overall mortality was improved by the addition of a panel of selected biomarkers [6]. Only plasma IL-6 single-handedly improved the ability to predict mortality.

The systemic inflammation is reflected by an increased number of neutrophil granulocytes in the circulation [2], and neutrophil granulocyte count is associated with progression of COPD [7]. Recently, neutrophil-to-lymphocyte ratio (NLR) has attracted attention as an inflammatory biomarker. It has been shown to be a prognostic biomarker in various inflammatory diseases, e.g. cardiovascular diseases [8,9], cancers [10,11] and psoriasis [12], and NLR is elevated in patients with COPD [13].

Calprotectin is mainly found in neutrophil granulocytes, where it comprises up to 45% of the cytosolic volume, and is thought to be secreted upon cell death or by a non-classical pathway [14-16]. It is a calcium binding heterodimer of S100A8/S100A9 which is believed to have both anti-inflammatory and antibacterial properties [17]. Calprotectin is elevated in a variety of inflammatory diseases [18-20], including respiratory diseases, and correlated with disease activity [21,22]. In patients suffering from cystic fibrosis, serum levels of calprotectin decreased in patients treated with antibiotics, and calprotectin levels measured at baseline and after treatment were inversely correlated with FEV_1_ and predicted time to next exacerbation [19]. Recently, we have shown that plasma calprotectin predicts all-cause mortality in patients with moderate to very severe COPD in stable phase [23].

Plasma concentration of YKL-40 (also named CHI3L1) is another potential biomarker of inflammatory activity in patients with COPD. Plasma YKL-40 is elevated, compared to normal levels, in patients with diseases hallmarked by chronic low-grade inflammation; cardiovascular diseases [24-26], cancer [27], liver fibrosis, inflammatory bowel disease and rheumatoid arthritis [28]. YKL-40 is secreted by activated macrophages and neutrophils and by cancer cells [29,30], and plays a role in inflammatory pulmonary disease. This glycoprotein is involved in cell proliferation and differentiation [31], inflammation [32], angiogenesis [33] and protects against apoptosis [34]. YKL-40 causes bronchial smooth cell proliferation and induces IL-8 expression from macrophages [35], as well as other mediators of inflammation [36]. IL-8 is an activator of neutrophil granulocytes. YKL-40 regulates cellular and tissue responses via IL-13 receptor α2 [37]. Plasma YKL-40 is linked to disease activity in patients with asthma, COPD and idiopathic pulmonary fibrosis, and high YKL-40 levels are found in bronchoalveolar lavage fluid from patients with pulmonary diseases [38-40]. We have recently demonstrated that plasma YKL-40 predict all-cause mortality in patients with moderate to very severe COPD in stable phase [41].

The aim of the present study was to investigate the correlation between plasma calprotectin and plasma YKL-40 and levels of neutrophil granulocytes, lymphocytes, and NLR in patients with moderate to very severe COPD in stable phase stratified by use of systemic glucocorticoids. We also studied the ability of these biomarkers to predict all-cause mortality in these two patient populations.

## Methods

### Study participants

575 patients diagnosed with COPD, and in a stable period of their disease, were in the period of 2001–2004 enrolled in a randomized controlled trial, which examined the effect of azithromycin 500 mg 3 days/month during a period of 36 months. Patients’ date of death was recorded in the Danish Central Registry and the last follow-up was January 31st 2011. After 10 years, only 0.9% of the patients had been lost to follow up.

Primary outcome measure was post broncho dilatator FEV_1_, and secondary outcome measures included quality of life, mortality, number of hospital admissions and days admitted in a hospital. The trial was registered at https://clinicaltrials.gov/ct2/show/NCT00132860?term=NCT00132860&rank=1. Ethical permission for the study was obtained from the Regional Scientific Ethical Committee for Southern Denmark (approval number 19990031).

Primary outcome in the present study was all-cause mortality. Inclusion criteria and exclusion criteria of the original study are shown in Table 1. Of the 575 patients enrolled, serum was available from 441 patients for measurement of plasma calprotectin and YKL-40. Treatment with systemic glucocorticoids within 14 days prior to inclusion in the study was identified retrospectively using patient files. We excluded 55 patients due to lacking or inadequate information about glucocorticoid treatment. The present study population was 386 patients; 302 patients were classified as not using systemic glucocorticoids within 14 days prior to inclusion, and 84 were classified as using glucocorticoids.

**Table 1:** **Inclusion- and exclusion criteria of the original study**

**Inclusion criteria**	**Exclusion criteria**
• Patients above 50 years with minimum 1 hospital admission caused by COPD with or without exacerbation within the last 2 years.	• End stage COPD with < 3 years expected survival (typically bedridden patients being dyspnoeic at rest).
• Current smoker or ex-smoker.	• Patients with other known respiratory infection.
• Post broncho dilatator FEV_1_ < 60% in stable condition (4 weeks after hospitalization).	• Patients with known pulmonary malignancy.
	• Patients with other pulmonary diseases than COPD.
• < 300 mL broncho dilatator reversibility in FEV_1_.	
	• Patients with immunodeficiency. However, patients treated with steroids can be included.
	• Patients with known hereditary disposition to lung infections (e.g. alpha-1-antitrypsindeficiency, cystic fibrosis or primary ciliary dyskinesia).
	• Patients receiving long-term antibiotic treatment.
	• Patients with known allergy or intolerance to Azithromycin.
	• Pregnant or breastfeeding women.
	• Manifest heart, liver or renal insufficiency.
	• Patients that, for reasons not stated above, are unlikely to be able to participate in a study period of 3 years.

### Plasma samples

Blood sampling was done at baseline at a time where patients were in a stable period of their disease. Samples were taken before the first dose of study medication was taken. Blood for EDTA plasma was centrifuged within 1 hour after sampling, and plasma samples were frozen and stored at −80°C until analysis. Plasma concentration of calprotectin was determined in duplicates using a commercially available ELISA kit (Hycult Biotech, Uden, NL). The measurement was done at the M 7641 department, Rigshospitalet, Copenhagen, Denmark.

Plasma concentration of YKL-40 was determined in duplicate by a commercially available ELISA kit (Quidel, Santa Clara, CA, USA). Plasma concentration of YKL-40 is stable for up to 16 years when frozen at −80°C degrees [42]. The plasma samples had one cycle of freeze-thaw before analysis. The reference interval for plasma YKL-40 was determined from a previous study of 3130 healthy subjects (1837 women, 1293 men, aged 21–84 years) from the Danish general population [42]. They had no known disease at the time of blood sampling in 1991–1994 and remained healthy and alive during the 16-year follow-up period. From this study, an age dependent correlation was found between age and plasma concentrations of YKL-40 and a formula has been extrapolated from this study, which we applied to our present study.

### Statistics

**No glucocorticoid use:** In this group of 302 patients, NLR was available from 280 patients, lymphocyte count from 282 patients, and neutrophil granulocyte count from 288 patients.

**Glucocorticoid use:** In this group of 84 patients, NLR was available from 70 patients, lymphocyte count from 71 patients, and neutrophil granulocyte count from 72 patients.

**Analysis:** The distribution of each biomarker was inspected visually by histograms. All biomarkers showed a violation of the normal distribution, and Spearman’s rank correlation coefficient was used to determine potential associations between plasma calprotectin and plasma YKL-40 and neutrophil granulocyte count, lymphocyte count and NLR.

Kaplan-Meier plots and univariate log-rank tests were computed for each biomarker. Each biomarker was dichotomized. The median of the non-glucocorticoid group was used to divide plasma calprotectin (135.53 ng/mL) and NLR (2.83). Lymphocyte count was divided by the lower limit of our reference value (1.3 × 10^9^/L). Neutrophil count was divided by the upper limit of our reference value (7.0 × 10^9^/L). Plasma YKL-40 was divided at the 75th age corrected percentile of the reference interval [42].

We performed a univariate Cox regression analysis to calculate hazard ratios (HR) and 95% confidence intervals (CI) for each biomarker as a continuous variable. Multivariate Cox regression was performed to control for confounders. Pre-analysis, the following parameters were defined to be included in the analysis: age (continuous covariate), gender, active smoking status at baseline, BMI (<20), Charlson Score Index (CSI >3) and GOLD-stage (defined as GOLD-stage 2 (inactive/moderate COPD): 79–50 FEV_1_% predicted; GOLD-stage 3 (severe COPD: 30–50 FEV_1_% predicted); and GOLD-stage 4 (very severe COPD: <30 FEV_1_% predicted)) [1]. Our Cox regression models did not include interactions. This was necessary in order to fit similar regression models for all biomarkers of interest, making the models comparable. Since our study population is small, we found it important to have a simple, transparent and replicable model, although it may increase the risk of overlooking a confounding interaction [43].

Proportional hazards (PH) assumption was tested using log-log plots and testing for time varying covariates using likelihood ratio test statistics. Due to violation of the PH assumption for GOLD-stage after an analysis time of 5 years, it was decided to restrict the Cox regression model to time ≤ 5 years. In this model we had 150 events (deaths) in the group not treated with systemic steroids, and 59 events in the steroid treated group. No violation of the PH assumption in the model restricted to time ≤ 5 years was found. Using likelihood ratio test statistics we excluded CSI > 3 as a confounder from all 5 regressions, as it did not contribute significantly to the analysis. Thus, we ended up with regression models for all 5 biomarkers adjusting for age, gender, active smoking status at baseline, BMI < 20 and GOLD-stage. Due to severe violations of the PH assumption for most covariates in the group using systemic glucocorticoids, we were unable to fit a multivariate Cox regression model in this group. All statistical analyses were carried out using Stata 13 (Stata Corp LP, TX, USA).

## Results

Characteristics of the study population stratified by use of systemic glucocorticoids are given in Table 2. The patients were characterized by having fairly advanced COPD, relatively high BMI and a high age. The group using systemic glucocorticoids had higher plasma calprotectin YKL-40, higher NLR and neutrophil granulocyte count, lower lymphocyte count, lower FEV_1_%-predicted and significantly shorter survival time compared to the group not treated with systemic glucocorticoids.

**Table 2:** **Baseline characteristics of the study population stratified by systemic glucocorticoid use**

	**No glucocorticoid use**	**Glucocorticoid use**	**P**
	**(n = 302)**	**(n = 84)**	
	**Median (IQR)**	**Median (IQR)**	
**Calprotectin (ng/mL)**	135.53 (97.84 – 193.46)	176.05 (123.64 – 234.20)	<0.01
**Neutrophil count (x 10** ^**9**^ **/L)**	5.88 (4.60 – 7.32)	8.47 (6.68 – 10.20)	<0.01
**Lymphocyte count (x 10** ^**9**^ **/L)**	1.92 (1.47 – 2.46)	1.43 (1.01 – 1.78)	<0.01
**NLR**	2.83 (2.03 – 4.57)	5.79 (4.17 – 9.66)	<0.01
**YKL-40 (ng/mL)**	77 (53 – 119)	105 (55 – 140)	0.059
**Age (years)**	71 (64 – 75)	70.5 (65 – 77.5)	0.305
**BMI**	24.22 (20.70 – 27.70)	24.17 (20.86 – 27.69)	0.914
**FEV**_**1**_ **% predicted**	40.20 (30.34 – 49.50)	34.84 (27.89 – 42.57)	<0.01
	**Median (CI 95%)**	**Median (CI 95%)**	
**Survival time (days)**	1851 (1632 – 2109)	1080 (680 – 1283)	<0.01
	**n (%)**	**n (%)**	
**Gender (male)**	148 (49)	43 (51)	0.723
**CSI (>3)**	56 (19)	20 (24)	0.283
**Current smoker**	121 (42)	32 (38)	0.744
**Moderate COPD**	86 (28)	18 (21)	
**Severe COPD**	150 (50)	42 (50)	
**Very severe COPD**	66 (22)	24 (29)	
		**n (Median dose)**	
**Maintenance glucocorticoid therapy**	-	73 (10 mg/day)	

P-values for continuous variables are calculated with Kruskal-Wallis test. P-values for dichotomous variables are calculated with chi-squared test. NLR: Neutrophil-to-lymphocyte ratio. BMI: Body Mass Index. FEV_1_% predicted: Forced expiratory volume in 1 second, % predicted. CSI: Charlson Score Index for comorbidity. Moderate COPD (GOLD-stage 2: 79–50 FEV_1_% predicted), severe COPD (GOLD-stage 3: 30–50 FEV_1_% predicted), and very severe COPD (GOLD-stage 4: <30 FEV_1_%predicted). CI 95%: 95% confidence interval. IQR: Inter quartile range.

### Patients not treated with glucocorticoids

**Figure 1:**
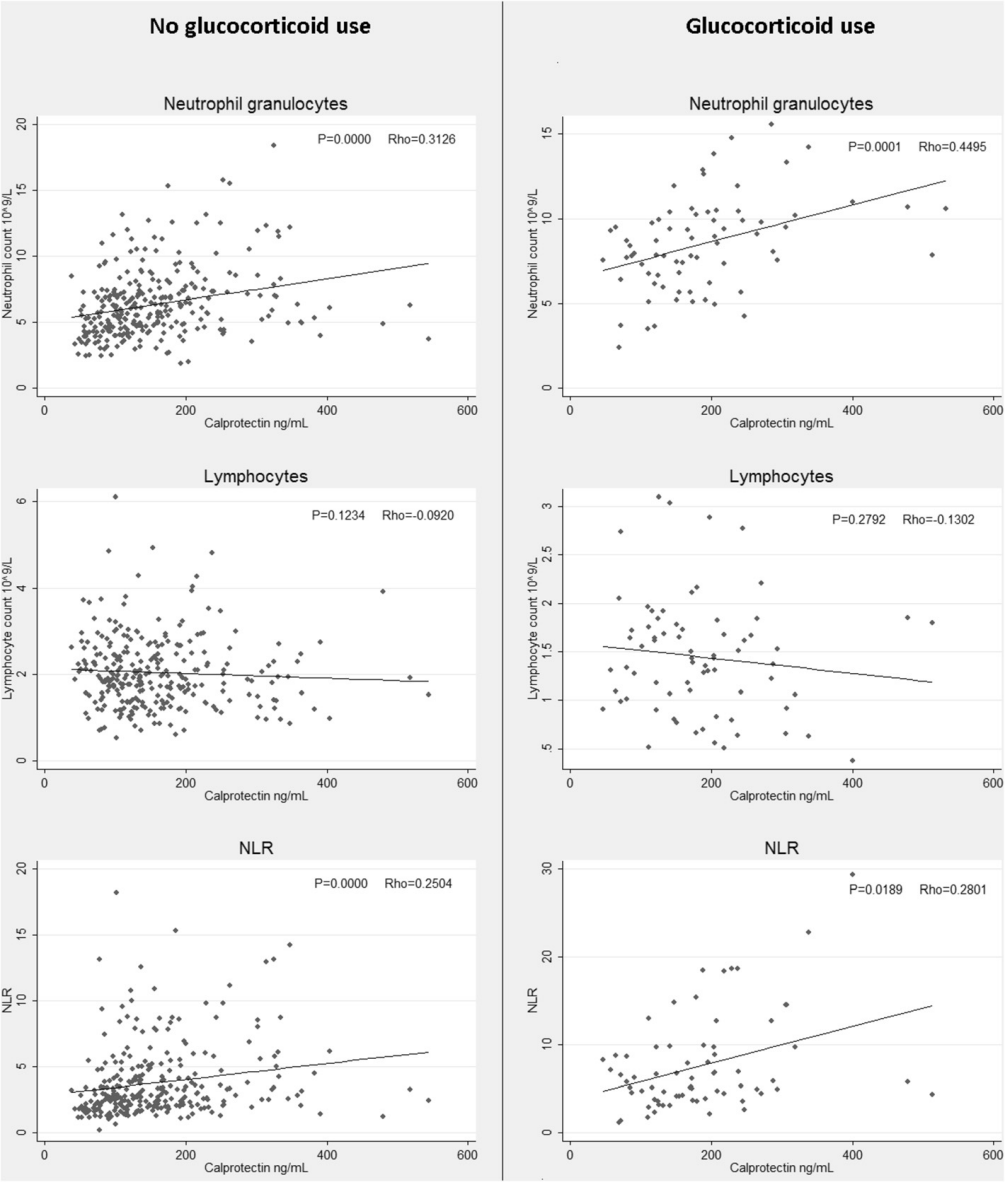
Relationship between calprotectin and neutrophil granulocyte count, lymphocyte count* and NLR stratified by glucocorticoid use. Spearman’s rank correlation coefficient (Rho) and corresponding p-values are displayed in the top right corner of each plot. *An outlier of lymphocytes = 30 × 10^9^/L was removed from the graph in the non-glucocorticoid group for aesthetic purposes.

**Figure 2:**
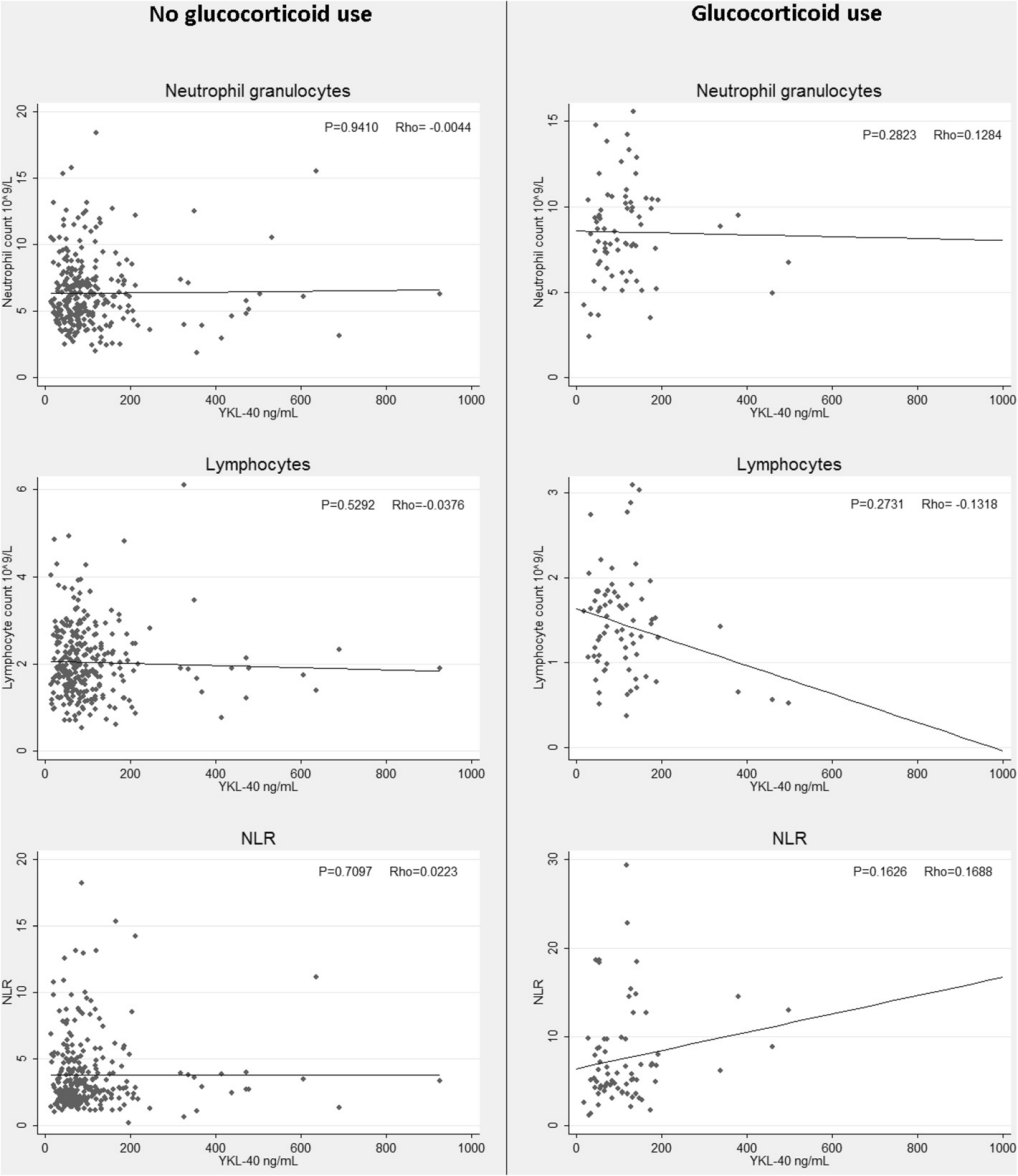
Relationship between YKL-40 and neutrophil granulocyte count, lymphocyte count* and NLR stratified by glucocorticoid use. Spearman’s rank correlation coefficient (Rho) and corresponding p-values are displayed in the top right corner of each plot. *An outlier of lymphocytes = 30 × 10^9^/L was removed from the graph in the non-glucocorticoid group for aesthetic purposes.

Figure 1 shows the correlation between plasma calprotectin and the three cellular biomarkers. A significant positive correlation was found between plasma calprotectin and neutrophil granulocyte count, and between plasma calprotectin and NLR. A negative correlation (but not statistically significant) was found between plasma calprotectin and lymphocyte count. There was no correlation between plasma YKL-40 and any of the cellular biomarkers (Figure 2).

**Figure 3:**
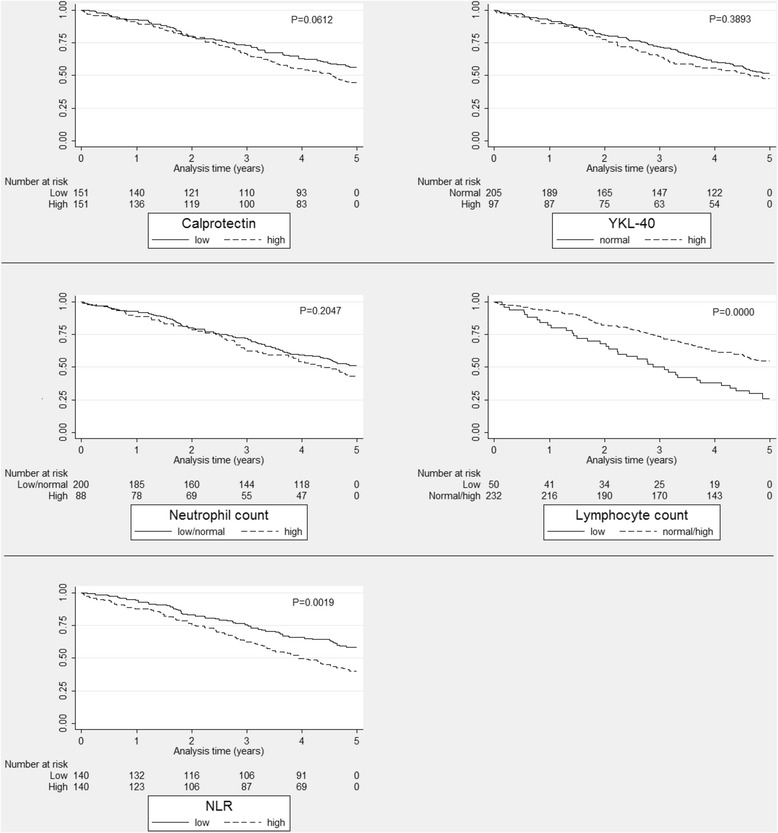
**Kaplan-Meier survival estimates for each biomarker in the group not treated with systemic glucocorticoids.** Calprotectin dichotomized at median (135.5 ng/mL). NLR dichotomized at median (2.83). Neutrophil count dichotomized at upper limit of reference value (7 × 10^9^/L). Lymphocyte count dichotomized at lower limit of reference value (1.3 × 10^9^/L). YKL-40 dichotomized at 75th age corrected percentile. NLR: Neutrophil-to-lymphocyte ratio.

Kaplan-Meier plots are shown in Figure 3. Low lymphocyte count and high NLR were significantly associated with higher mortality. No significant difference in mortality was found between the levels of plasma calprotectin, plasma YKL-40 and neutrophil granulocyte count. Univariate regression analysis showed that plasma calprotectin, NLR and lymphocyte count were predictors of mortality, whereas plasma YKL-40 and neutrophil granulocyte count were not. When adjusting for possible confounders, plasma calprotectin, NLR, and lymphocyte count were significantly associated with mortality (Table 3).

**Table 3:** **Results of uni- and multivariate Cox regressions for each biomarker restricted to time ≤ 5 years**

**No glucocorticoid use**				
	Unadjusted*		Adjusted**	
	**HR (CI 95%)**	**P**	**HR (CI 95%)**	**P**
**Calprotectin**	1.002 (1.000 – 1.004)	0.031	1.002 (1.000 – 1.004)	0.016
**Neutrophil count**	1.039 (0.978 – 1.103)	0.213	1.035 (0.971 – 1.103)	0.296
**Lymphocyte count**	0.604 (0.475 – 0.768)	<0.01	0.667 (0.522 – 0.851)	<0.01
**NLR**	1.104 (1.051 – 1.159)	<0.01	1.090 (1.036 – 1.148)	<0.01
**YKL-40**	1.001 (0.999 – 1.002)	0.318	1.001 (1.000 – 1.003)	0.086
**Glucocorticoid use**	Unadjusted*			
	**HR (CI 95%)**	**P**		
**Calprotectin**	1.001 (0.998 – 1.003)	0.559		
**Neutrophil count**	1.035 (0.945 – 1.133)	0.462		
**Lymphocyte count**	0.946 (0.580 – 1.545)	0.825		
**NLR**	1.010 (0.966 – 1.056)	0.665		
**YKL-40**	1.006 (1.003 – 1.008)	<0.01		

*Univariate Cox regression. ** Multivariate Cox regression adjusting for gender, age at baseline, GOLD-stage, smoking status at baseline and BMI < 20. NLR: Neutrophil-to-lymphocyte ratio. HR: Hazard ratio. CI 95%: 95% confidence interval.

### Patients treated with glucocorticoids

84 patients were treated with systemic glucocorticoids; 73 patients were on a long term maintenance therapy (median daily dose of 10 mg, range 5–40 mg) and 11 patients were classified as not having ended a high dose treatment within 14 days prior to inclusion (Table 2). There was a significant positive correlation between plasma calprotectin and neutrophil granulocyte count and NLR (Figure 1). No significant correlations were found between plasma YKL-40 and the cellular biomarkers (Figure 2).

**Figure 4:**
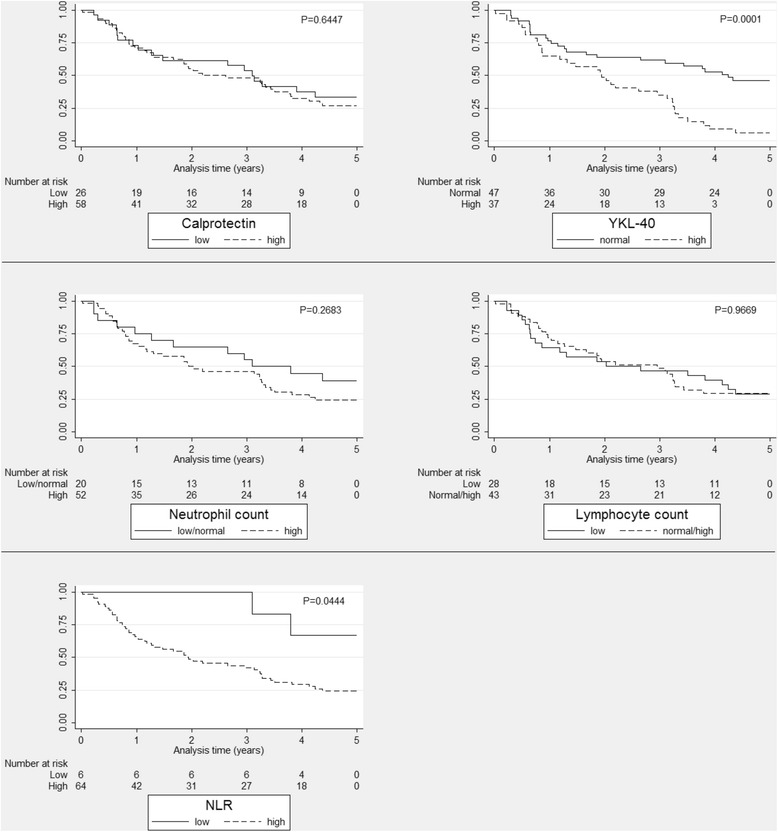
**Kaplan-Meier survival estimates for each biomarker in the group treated with systemic glucocorticoids.** Calprotectin dichotomized at median of non-glucocorticoid group (135.5 ng/mL). NLR dichotomized at median of non-glucocorticoid group (2.83). Neutrophil granulocyte count dichotomized at upper limit of reference value (7 × 10^9^/L). Lymphocyte count dichotomized at lower limit of reference value (1.3 × 10^9^/L). YKL-40 dichotomized at 75th age corrected percentile. NLR: Neutrophil-to-lymphocyte ratio.

Kaplan-Meier plots are shown in Figure 4. High plasma YKL-40 level was associated with shorter overall survival. Univariate cox regression analysis showed that plasma YKL-40 was significantly associated with mortality (Table 3).

## Discussion

We investigated potential cellular and plasma biomarkers of inflammatory activity in two populations of patients with moderate to severe COPD. One did not receive treatment with systemic glucocorticoids and one group received or had recently been treated with systemic glucocorticoids. We found that plasma calprotectin was positively correlated with neutrophil granulocyte count and NLR. These correlations appear attenuated in the group treated with systemic glucocorticoids. No significant associations were found between plasma YKL-40 and the cellular biomarkers. High plasma calprotectin, low lymphocyte count and high NLR were independent predictors of all-cause mortality in patients not treated with glucocorticoids. On the other hand, high plasma YKL-40 was associated with increased mortality in the glucocorticoid treated group.

As expected, a positive correlation was found between plasma calprotectin and neutrophil granulocyte count. However, this does not mean that plasma calprotectin is just a reflection of total blood neutrophil count. Calprotectin is released almost entirely from dead neutrophil granulocytes and neutrophil granulocytes with disrupted membranes [14,15]. Plasma calprotectin levels likely reflect the number of neutrophil granulocytes currently participating in inflammatory activity, and not just their total number. As neutrophil granulocyte activity is related to airway inflammation in patients with COPD [44], our finding adds evidence to the hypothesis that plasma calprotectin may be a biomarker of airway inflammation in patients with COPD.

In the glucocorticoid treated group, there was a significantly lower lymphocyte count and higher plasma calprotectin and YKL-40, higher neutrophil granulocyte count and higher NLR. This may be explained partly by an effect of glucocorticoids and partly by more advanced disease in the glucocorticoid group. Systemic glucocorticoids cause lymphopenia [45] and neutrophilocytosis [46], and consequently NLR will increase in patients treated with glucocorticoids. The patients treated with systemic glucocorticoids had significantly lower mean FEV_1_% predicted than the non-treated patients, suggesting that they had more advanced or more aggressive disease, and that the difference in biomarker levels to some degree could reflect these differences (Table 2). Patients in the glucocorticoid treated group may be characterized as a systemic inflammatory phenotype as proposed by Agusti et al. [5].

In the glucocorticoid treated group, plasma calprotectin was positively correlated with neutrophil granulocyte count and NLR, whereas plasma YKL-40 was not correlated with any of the cellular biomarkers. We found no noticeable differences in correlations between plasma biomarkers and cellular biomarkers between the two patient groups.

The monitoring of systemic inflammatory biomarker blood levels may potentially provide an additional level of risk stratification in patients with COPD. Useful biomarkers that reflect disease severity and respond to treatment are receiving increasing attention [47]. Usage of the conventional biomarker, C-reactive protein, in risk management of COPD is hampered by inconsistent results and a high degree of variability [48-50].

To our knowledge, this is the first study to investigate the usefulness of leukocyte subpopulations in the prediction of mortality in patients with moderate to very severe COPD. We found that low lymphocyte count was a significant predictor of increased mortality. A growing interest has been on lymphopenia and NLR as predictors of mortality in various clinical settings. Low lymphocyte count is associated with poor outcomes in patients suffering from acute medical conditions such as sepsis [51], bacteremia [52] and trauma [53], and also in patients with chronic diseases such as cardiovascular diseases [54], cancers [55] and inflammatory bowel disease [56]. The mechanism of lymphopenia in critically ill patients involve apoptosis and redistribution of lymphocytes [57,58], but little is known about the causes of lymphopenia in patients with chronic inflammatory diseases. Lymphopenia is associated with age [59] and poor nutritional status [60], which also characterizes COPD [61].These factors were adjusted for in our regression models. Lymphopenia appears to be a biomarker of poor overall survival in general, and not specific to COPD.

In contrast, we found no association between neutrophil granulocyte count and mortality. This is surprising since neutrophil granulocyte count has previously been described as a predictor of mortality [6]. The statistical power of our study may not have been sufficient to detect such associations. The reason for the development of neutrophilocytosis in patients with COPD is not fully understood, but it is hypothesized to be a result of “overspill” from the airway inflammation that characterizes COPD [2].

NLR, the simple ratio obtained from a differential blood cell count, was associated with mortality in our study. NLR provides information beyond that of a complete white blood cell count (high neutrophil granulocyte count reflecting systemic inflammation and lymphopenia reflecting immune competence).

We have previously shown that plasma calprotectin and plasma YKL-40 were independent predictors of mortality in this same cohort of patients without stratifying for systemic glucocorticoid use [23,41]. In the present study, plasma calprotectin was a significant biomarker of mortality in the patients not treated with glucocorticoids, whereas plasma YKL-40 was not. Our results suggest that calprotectin identifies a subgroup of patients with higher mortality.

In the patients treated with glucocorticoids, plasma calprotectin, neutrophil granulocyte count, lymphocyte count and NLR were not associated with mortality, whereas higher plasma YKL-40 was a predictor of mortality. This group included only 84 patients, and despite 59 fatalities the statistical power may not have been strong enough to detect associations with the other markers.

An important pharmacological effect of systemic glucocorticoids is that they may cause significant neutrophilocytosis and lymphopenia. This may explain why neutrophil count, lymphocyte count and NLR do not act as markers of mortality in this group of patients. The effect of glucocorticoids in patients with COPD on plasma calprotectin and YKL-40 levels has not previously been described. S100A8 and S100A9 expression in mice is inhibited by glucocorticoids [62]. Since systemic glucocorticoids induce neutrophilocytosis and plasma calprotectin level is positively correlated with neutrophil granulocyte count, we hypothesize that though glucocorticoids may inhibit expression of calprotectin in humans, the effect of neutrophilocytocis is more profound than this inhibition. Consequently, plasma calprotectin will increase with systemic glucocorticoid treatment, and like the cellular markers lose its ability to be a predictor of mortality. This may explain why plasma calprotectin level is not a marker of mortality in this group.

Treatment with glucocorticoids in combination with disease modifying anti-rheumatic drugs decreases serum YKL-40 in patients with RA [63]. This decrease is probably a reflection of decreased inflammatory activity, and not a direct effect on YKL-40. In our study, plasma YKL-40 was the only significant predictor of all-cause mortality in the COPD patients treated with systemic glucocorticoids, whereas it was non-significant in the group not using systemic glucocorticoids. Plasma YKL-40 may be a better predictor of mortality in patients with more severe inflammation than in patients with low-grade basal inflammation. Although the exact role of YKL-40 in the pathogenesis of COPD is unknown, given its characteristics it can be speculated that it is more than a biomarker reflecting inflammation, but rather an active player in the inflammatory cascade. A recent study has demonstrated that anti-YKL-40 antibody therapy inhibits tumor vascularization and progression in mice with glioblastoma [64]. This places YKL-40 as a potential therapeutic target, which may not only be confined to cancer diseases, but also to inflammatory diseases.

The finding of a difference in the ability of inflammatory biomarkers to predict mortality between COPD patients treated with or without systemic glucocorticoids presents new challenges. Although this difference may not be due to systemic glucocorticoid treatment, it is an important question to address. Regular treatment with systemic glucocorticoids in patients with COPD is now obsolete, but the momentary use of systemic glucocorticoids is a key treatment in many diseases. Therefore, studies are called for to investigate the effect of systemic glucocorticoids on inflammatory biomarkers and their ability to predict poor survival.

Our study has some limitations. Data regarding systemic glucocorticoid treatment was collected retrospectively, and the indications for their use were in most cases unknown to us. The use of long term systemic treatment with glucocorticoids was probably mostly used in patients with many and severe episodes of acute exacerbation, meaning that the group of patients in glucocorticoid treatment may be significantly different from the group that did not receive glucocorticoids. Therefore, direct comparison between the group should not be made. Another limitation is that our cohort was small when compared to other survival studies in patients with COPD [65,66]. This was to some degree balanced out by a high number of outcomes (150 deaths is well within the 10 per covariate as suggested by Peduzzi et al. [67]). Strengths of our study were the well-defined study population, and the long follow-up time with almost complete follow-up.

In conclusion, we have shown that plasma calprotectin is correlated with neutrophil granulocyte count and NLR in patients with moderate to very severe stable COPD not treated with systemic glucocorticoids. Furthermore, plasma calprotectin, NLR and lymphocyte count were independent predictors of all-cause mortality in these patients. We also showed that treatment with systemic glucocorticoids may have an impact on the ability of biomarkers to predict all-cause mortality. Validation studies are needed to clarify the effect of glucocorticoid treatment on these biomarkers and their ability to predict adverse outcomes, and whether some of these biomarkers will improve the current clinical prediction of mortality in patients with COPD.
